# Two novel sequence variants in *MSH2* gene in a patient who underwent cancer genetic counseling for a very early-onset epithelial ovarian cancer

**DOI:** 10.1186/s13053-016-0054-5

**Published:** 2016-09-06

**Authors:** Matilde Pensabene, Caterina Condello, Chiara Carlomagno, Sabino De Placido, Raffaella Liccardo, Francesca Duraturo

**Affiliations:** 1 Department of Clinical Medicine – Clinical Unit of Oncology, University Hospital Federico II, Naples, Italy; 2 Department of Clinical Medicine and Surgery, University Federico II, Naples, Italy; 3 Department of Molecular Medicine and Medical Biotechnology, University Federico II, Naples, Italy

**Keywords:** MSH2 germ-line mutation, Cancer genetic counseling, Hereditary ovarian cancer, Lynch syndrome

## Abstract

**Background:**

Early-onset or hereditary ovarian cancer is mostly associated with *BRCA1* or *BRCA2* mutations. Mismatch repair genes sequence alteration frequently cause colorectal cancer, and, in less extent, other tumors, such as ovarian cancer. Subjects with personal and/or family history suggestive for hereditary cancer should be addressed to cancer genetic counseling, aimed to the identification, definition and management of hereditary cancer syndrome, by a multidisciplinary approach.

**Case presentation:**

A woman with a very early onset epithelial ovarian cancer underwent to cancer genetic counseling and genetic testing. Pedigree analysis suggested a clinical diagnosis of Lynch II syndrome, according to the Amsterdam criteria. The MMRpro model showed a cumulative risk of mutation of 50.3 %, thus, genetic testing was offered to the patient. Two germ-line mutations have been identified in exon 11 of *MSH2* gene: c.1706A > T (p.Glu569Val) and c.1711G > T (p.Glu571*). Both DNA alterations were novel mutations not yet described in literature. The first is a missense mutation that is to be considered an unclassified variant; the second is nonsense mutation that created a premature stop codon resulting in a truncated not functioning protein. Both genetic alterations were found in the patients’ father DNA.

**Conclusions:**

The present report finds out two unpublished sequence alterations in exon 11 of the *MSH2* gene, one on which can be considered causative of Lynch phenotype. Moreover, it stresses the importance of the multidisciplinary onco-genetic counselling in order to correctly frame the hereditary syndrome, suggest the right genetic test, and offer the most appropriate management of the cancer risk for the patients and her family members.

## Background

Progresses in the molecular genetics of cancer have led to the identification of genes predisposing for hereditary ovarian cancer [1]. About 84 % of hereditary ovarian cancers derive from *BRCA1* or *BRCA2* mutations that sustain the hereditary breast/ovarian cancer (HBOC) syndrome. The overall risk of developing ovarian cancer is about 49 % for *BRCA1* and 11 % for *BRCA2* mutated subjects, and the mean age at diagnosis is 50.8 years [2]. Few cases (about 2 %) of hereditary ovarian cancer derive from mutation in mismatch repair (MMR) genes that sustain the Lynch syndrome [3]. Carriers of *MLH1/MSH2* or *MSH6* genes mutations have an overall risk of developing ovarian cancer of about 4–24 and 1–11 %, respectively. The mean age at diagnosis of ovarian cancer for these patients is 42 and 46 years, respectively.

So far, numerous sequence variants listed in the international database [4] have been identified in MMR genes (*MLH1, MSH2, MSH6, PMS2, MSH3*). The majority of mutations in the MMR genes identified are missense, nonsense or small insertions/deletions. Moreover, large genomic rearrangements can predispose to Lynch syndrome [5].

Given the complexity of issues related to hereditary/familial cancers and clinical implications of genetic testing, cancer genetic counseling actually is the most suitable approach to manage high risk subjects. There is general consensus that the management of hereditary and familial cancer requires a multidisciplinary approach [6, 7]. The counseling model used at our Clinical Unit foresees close links with the oncologist, psychologist, geneticist and other professionals [8].

Here we report a case in which mutational analysis was performed, within cancer genetic counseling, and revealed two novel sequence variants in the *MSH2* gene, not listed in the SNP database.

## Methods

### Cancer genetic counseling

Patients or disease-free subjects with a suspected familiar or hereditary tumors are referred to the Hereditary and Familiar Tumor Unit at the Department of Clinical Medicine of the University Hospital Federico II in Naples. Cancer genetic counseling is performed by a multidisciplinary team including dedicated oncologist and psychologist, according to the multistep model designed and validated within the Italian Network for “Hereditary Breast and/or Ovarian Cancer”. Structured sessions with a psycho-oncologist for psychological assessment, psychological counseling interventions and psychotherapy or emotional support are also planned for patients and their at-risk subjects [6, 9]. During the cancer genetic counseling steps devoted to information-giving and pedigree construction, anxiety and depression levels were assessed from replies to self-report questionnaire administered by the psycho-oncologist. The Hospital and Depression Scale (HAD Scale) were used during psychological interview to determine the presence or not of distress [10].

The hereditary risk is calculated according by clinical and probabilistic tools, such as the Amsterdam and/or Bethesda criteria and MMRpro model. When clinical criteria are satisfied and/or an *a priori* hereditary risk is ≥10 %, the genetic testing for the MMR genes is offered to the affected subjects [11, 12]. Preventive measures are offered on the basis of risk profile and genetic test results according with NCCN guidelines.

Written informed consent was obtained for publication of this case.

### Mutational analysis

Total genomic DNA was extracted from 4 mL peripheral blood lymphocytes of the patient and her parents using a BACC2 Nucleon Kit (GE Healthcare). All MLH1, and MSH2 exons were amplified, including intron-exon boundaries, using customized primer sets. A Transgenomic Wave DNA Fragment Analysis System (3500 HT, Transgenomic Inc., Omaha, Nebraska, USA) was used to perform denaturing High Performance Liquid Chromatography (dHPLC) analysis. Abnormal elution profiles were identified by visual inspection of the chromatogram on the basis of the appearance of one or more additional earlier eluting peaks. For abnormal dHPLC profiles, genomic DNA of the patient was re-amplified and sequenced in both the forward and reverse directions using an ABI 3100 Genetic Analyser (Applied Biosystems, Foster City, Ca., USA).

Total RNA was extracted from the lymphocytes of the patient and three normal controls by using Trizol reagent (Invitrogen, Life Technologies, Ca, USA). A total of 1 microgram of RNA from each sample was retro-transcribed. cDNA was synthesized using 1 μg of total RNA, 500 ng of random hexamers and 1 μl Superscript III reverse transcriptase (Invitrogen, Life Technologies, Ca, USA), in the presence of 4 μl 5X RT buffer, 1 μl DTT (0.1 M) and 1 mM dNTPs. The reaction was run for 50 min at 42 °C in a 20 μl reaction volume, heated to 70 °C for 15 min and quick chilled on ice.

For *MSH2* analysis, cDNA was amplified using the forward primer overlapping the junction between exons 9–10 (5′-CCAGAGATCTTGGCTTGGAC-3′) and the reverse primer located in exon 15 (5′-AGCACTTCTTTGCTGCTGGT-3′). The amplified fragment was visualized on an 8 % polyacrilamide gel. Anomalous bands were excised from the gel and re-suspended in H_2_O. The bands were then re-amplified using the same forward primer and a reverse primer in exon 13 (5′-CTCACATGGCACAAAACACC-3′); subsequently, these fragments were sequenced.

### Microsatellite instability assay

Microsatellite instability (MSI) was tested on paired samples from peripheral blood lymphocytes and paraffin-embedded tumor ovarian tissues. The MSI status was evaluated with a fluorescent multiplex system comprising 6 mononucleotide repeats (BAT-40, BAT-25, BAT-26, NR-21, NR-24 and NR-27), 4 dinucleotide repeats (D2S123, D5S346, D18S58 and D17S250) and 2 tetranucleotide repeats (TPOX and THO1) as biochemical markers of individuality using the CC-MSI Kit (AbAnalitica, Padova, Italy) and subsequent capillary electrophoresis analysis using an ABI 3130 Prism (Applied Biosystems). Tumors were classified as “highly unstable” (MSI-H), if ≥ 30 % of the markers showed instabilities and as “low unstable” (MSI-L), if ≥ 10 % of the markers showed instabilities; if no allele difference between DNA extracted by normal and tumoral tissues was observed, tumors were classified as microsatellite-stable (MSS).

### Immunohistochemistry analysis

The expression levels of MSH2, MLH1 and MSH6 proteins were measured by immunohistochemical (IHC) analysis on paraffin-embedded tissue sections of ovarian cancer from our patient. The IHC analysis was performed on a Benchmark XT automatized immunostainer (Ventana Medical Biosystems, Tucson, USA). The antibody used were anti-MSH6, mouse monoclonal clone 44, anti-MSH2, mouse monoclonal clone G219-1129, and anti-MLH1, mouse monoclonal clone M1 (Ventana). The detection system used was an iVIEW DAB Detection Kit (Ventana) which is based on the Streptavidin-Biotin-conjugated revelation system. Nuclear staining was observed with an optical microscope with positivity represented by the presence of brown staining. This positivity was compared to blue nuclear epitopes, in which the specific antigen was not present. The internal positive control was represented by lymphocytes, stroma and functional mucosal crypts, while the negative control was obtained by slides without primary antibody. Nuclear immunoreactivity scores were assigned using range from 0 to 100 %.

## Case presentation

A 29-year-old woman received diagnosis of clear cell ovarian adenocarcinoma, stage IA according to the International Federation of Gynecology and Obstetrics classification, and underwent radical surgery, followed by six cycles of carboplatin and paclitaxel. She requested counseling because of a very early-onset ovarian carcinoma and a familial clustering of colon cancer both on maternal and paternal lines.

**Fig. 1:**
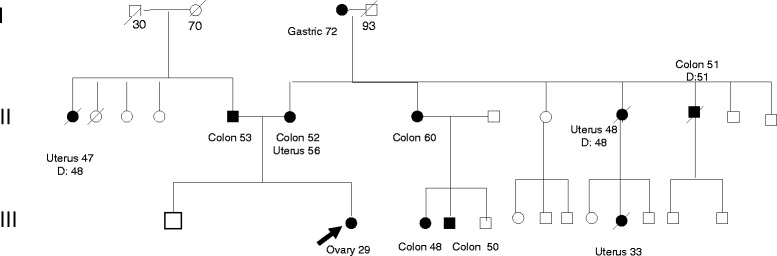
Patient’s pedigree. The arrow indicates the early-onset ovarian cancer proband. Filled symbols indicate subjects affected by cancer; square = male; circle = female. The numbers after cancer sites indicate the age at diagnosis. The age of death is reported if known. Abbreviations: D = deceased

Pedigree analysis revealed one case of colon cancer and one of uterine cancer on paternal line, and four colon cancers, one endometrial cancer, and one both colon and endometrial cancer on maternal line (Fig. 1).

Clinical diagnosis of Lynch II syndrome was done according to the Amsterdam criteria [12]. The MMRpro [11] model showed a cumulative risk of mutation of 50.3 % (22 % for *hMLH1*, 24.3 % for *hMSH2*, and 6.6 % for hMSH6), thus, genetic testing was offered to the patient.

Mutation detection was performed on *MLH1* and *MSH2* coding regions of the index case and her parents. The patient was found to be heterozygous for two sequence alterations (c.1706A > T and c.1711G > T), both in exon 11 of *MSH2* gene. The first mutation predicted a substitution of a glutamate residue with a valine residue at position 569 (p.Glu569Val); the second mutation created a premature stop of codon transcription, resulting in a truncated protein (p.Glu571*). Both are novel genetic variants not yet described in literature.

**Fig. 2:**
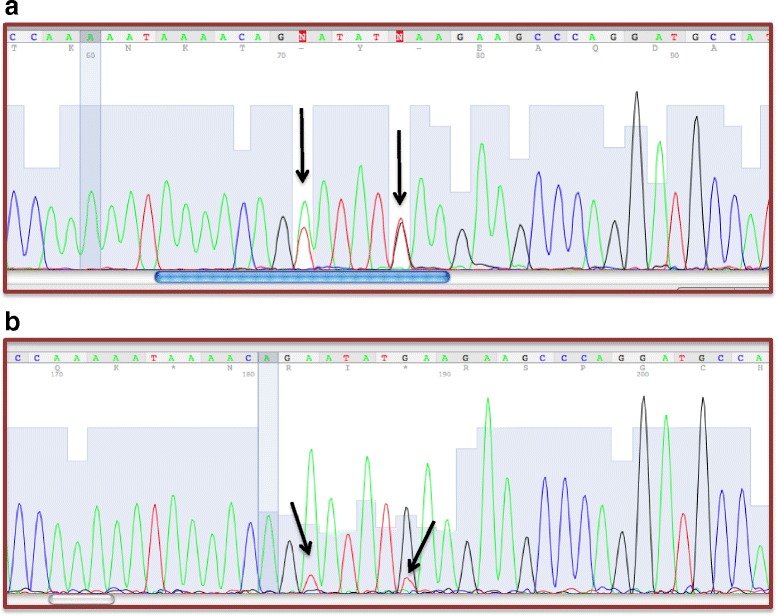
The heterozygous state for the mutations (c.1706A > T) and (c.1711G > T) at exon 11 of MSH2 gene. **a** MSH2 genomic sequence analysis (fragment including exon 11) showing the two mutations identified in our index case. The arrow indicates the position of two mutations. **b** MSH2 cDNA sequence analysis (fragment including exons 9–15) showing the two mutations at mRNA level. The arrow indicates the lowering peak of mutant allele

**Fig. 3:**
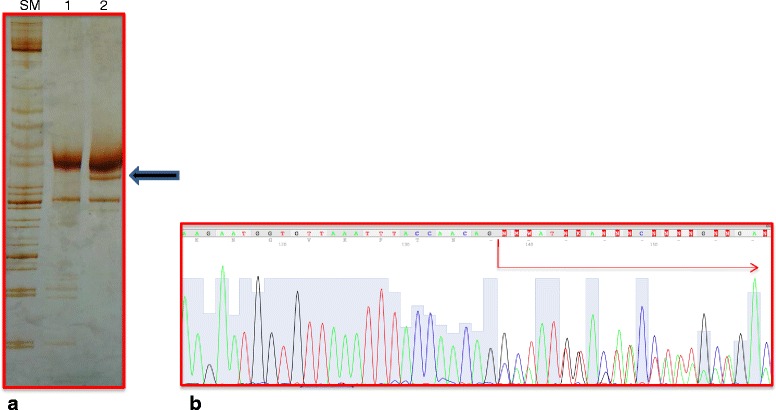
Characterization of aberrant splicing fragment. **a** 8 % polyacrilamide gel electrophoresis of MSH2 cDNA RT-PCR analysis of fragment in a normal control (1) and in the index case (2). SM size marker XIV Roche. The arrow indicates the abnormal band lower than the wild type band (1172 bp) of about 1000 bp. **b** MSH2 cDNA sequence analysis of abnormal band showing the skipping of exon 11. The arrow indicates the frameshift (between 11 and 12 exons) due to contamination with wild type allele

Direct sequencing of *MSH2* mRNA transcript including exon 9–15 revealed the presence of only the wild type allele (Fig. 2). Moreover, electrophoresis of this transcript showed an abnormal fragment, suggestive of an aberrant splicing event. Direct sequencing of this aberrantly spliced transcript revealed the loss in frame of the entire exon 11; this transcript fragment resulted in a new exon connection between 10 and 12 exons (Fig. 3).

The same two genetic alterations were found in the patients’ father DNA.

Genetic testing performed on DNA from patient’s mother revealed a pathogenic mutation, c.229 T > C, in exon 3 of the *MLH1* gene, which determines a dramatic amino-acid change at protein level, p.Cys77Arg. This mutation was not identified in our index case.

*BRCA1* and *BRCA2* genes were also analysed, and no mutation was found.

Instability in three microsatellite markers (BAT40, D18S58 and D2S123) out of ten was found thus, ovarian cancer tissue from our proband showed a MSI-H status.

**Fig. 4:**
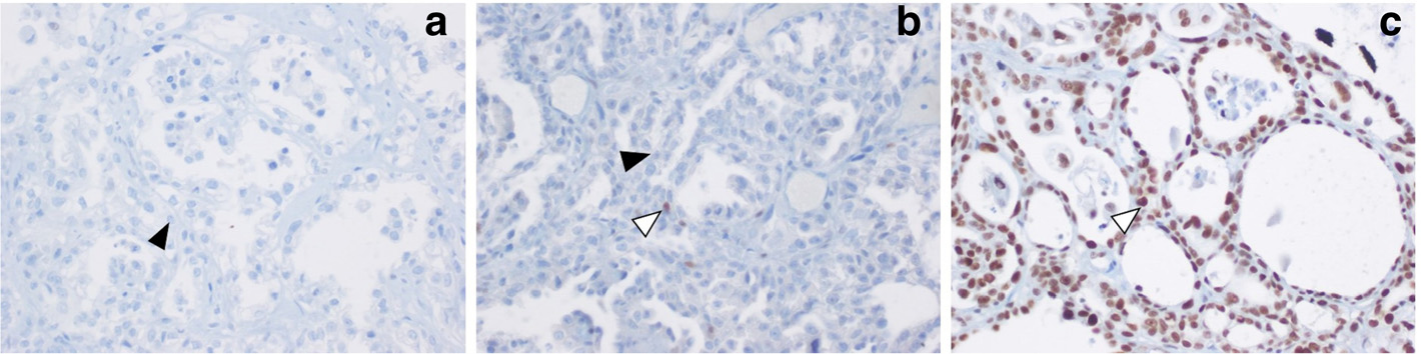
MSH2, MSH6 and MLH1 immunohistochemistry (IHC) results in the ovary tumor section of patient carrier of c.1706A > T and c.1711G > T variants in MSH2 exon 11. **a** Absence of MSH2 protein in the tumor cells; **b** weak presence of MSH6 protein in the tumor cells; **c** normal IHC for MLH1 protein in the tumor cells. Filled arrow heads point to IHC– tumor cells, open arrows heads point to IHC+ tumor cells. Blue nuclear staining of lymphocytes indicates positive internal control

The IHC analysis revealed loss of expression of MSH2/MSH6 protein complex on the tissue sections of ovarian cancer from our patient (Fig. 4).

In post-test counseling, the patient was informed about the results of the genetic test and their clinical implications. A surveillance program was proposed, including yearly colonoscopy and urinary cytology, according with NCCN guidelines for *MSH2* gene mutation carriers, and breast ultrasound and magnetic resonance, because of her young age, and complex breast parenchyma.

The HAD Scale revealed moderate levels of anxiety and depression, and the psychological interview showed a significant concern for the hereditary risk and an experience of suffering for the foreclosure of motherhood because of bilateral oophorectomy. Cancer genetic counselling had a positive impact on perception of control over important life events, such as experience of cancer disease and physical and psychic sequelae. It promoted also a positive reappraisal of chances and goals. At the conclusion of counseling, indeed, the patient has adhered to the intensified surveillance program, and chose to consider the possibility of an adoption path for a child with her husband.

## Discussion

Cancer genetic counseling should be always offered during the work-up of young patients with epithelial ovarian cancer, in order to screen the presence of a hereditary syndrome and to improve the oncological follow-up including intensified surveillance. Clinicians should be particularly careful to the evaluation of a positive family history of cancer, being ovarian cancer involved in three different syndromes, such as site-specific, HBOC and Lynch Syndrome.

The proper placement of the present case within Lynch syndrome frame has been very helpful to direct appropriately the genetic testing, and the surveillance planning. In fact, *MSH2* gene mutation carriers are known to have an increased risk for cancer in different body sites, such as colon, breast and genitourinary tract.

Genetic testing of our patient’s DNA showed two sequence alterations in exon 11 of the MSH2 gene. So far, both the missense (c.1706A > T) and the nonsense (c.1711G > T) mutations are not described in literature. Parental testing revealed that both mutations were of paternal origin, and they were on the same allele. The nonsense mutation determines a premature stop codon (p.Glu571*), therefore, it has a clear functional role, because it creates a truncated protein, thus, it can be considered causative of Lynch phenotype. The missense mutation (c.1706A > T) should result in an amino acid change at the protein level (p.Glu569Val). A large number of genetic alterations that are still classified as “variants of unknown significance” have pathogenic effects on the mRNA splicing process [13]. In the present case, the missense mutation could activate a cryptic splicing site. Indeed, the electrophoresis of MSH2 cDNA amplified fragment showed an abnormal band which was found to correspond to an aberrantly spliced transcript, with the loss of exon 11. This transcript showed a new junction between exons 10–12, and it should create a premature stop codon in exon 12 (9 codons next the new junction). Interestingly, MSH2 cDNA sequencing analysis showed a dramatic reduction of the signal corresponding to the mutated transcript with respect to the wild type allele. Therefore, it is conceivable that a nonsense-mediated decay (NMD) mechanism [14] of transcripts bearing the nonsense c.1711G > T mutation or, alternatively, transcripts missing exon 11 due to the c.1706A > T missense mutation is responsible for the reduced levels of the mutated allele. To our best of knowledge this is the first report of co-existence of two putative functional mutations on a single MSH2 allele; both these variants create truncated transcripts that are prematurely degraded. Although, we can not speculate that these two variants have a synergistic effect on Lynch phenotype, we can likely conclude that these mutations in MSH2 gene are responsible of proband’s phenotype. Indeed, IHC data showed the loss expression of MSH2 and MSH6 proteins on ovarian cancer tissue from our index case and the somatic DNA extracted from this tissue also showed a status MSI-H; it was compatible with genotype of our proband.

Finally, the index case was not carrier of mutation in MLH1 gene identified in her mother. The early onset of ovarian cancer, that is an infrequent tumor in Lynch syndrome, could rather be explained by the presence of modifier genes of phenotype [15].

Hereditary ovarian cancer involves a complex array of medical and psychological aspects that impact on affected subjects and their family members. The anxiety and depression levels showed by the patient were in relation to the threat of hereditary cancer risk and the sequelae of oncological treatments [16]. The present case confirms that cancer genetic counselling is a successful modality in the management of at-risk subjects by a multidisciplinary team, including psycho-oncologist. The space of emotional containment offered by the cancer genetic counselling has provided a sense of safety for the patient to explore feelings that were experienced as overwhelming and confusing.

## Conclusions

The identification of novel mutations with a clear pathogenic effect is a very important finding, because it allows to understand better the onset of cancers in very young subjects, to offer the most appropriate cancer genetic counselling and cancer risk management to the patient and her family members.

The present report also emphasizes that the multidisciplinary approach is the most appropriate way to take care of a young patient with ovarian cancer, in order to manage the complex issues related to ovarian cancer diagnosis, long-term sequelae and genetic predisposition to cancer.

## Abbreviations

dHPLC, denaturing High Performance Liquid Chromatography; dNTPs, Deoxyribonucleoside-triphosphates; DTT, dithiothreitol; FIGO, International Federation of Gynecology and Obstetrics; HAD Scale, Hospital and Depression Scale; HBOC, hereditary breast/ovarian cancer; MMR, mismatch repair; MMRpro, mismatch repair probability; MSI, microsatellite instability; MSI-H, microsatellite instability high; MSI-L, microsatellite instability low; MSS, microsatellite-stable; NCCN, National Comprehensive Cancer Network; NMD, nonsense-mediated decay; RT, reverse transcriptase; SNP, Single Nucleotide Polymorphism
